# Development and validation of a predicative model for identifying sarcopenia in Chinese adults using nutrition indicators (AHLC)

**DOI:** 10.3389/fnut.2024.1505655

**Published:** 2024-12-12

**Authors:** Xin Zhao, Pengdong Yan, Ningxin Chen, Tingting Han, Bin Wang, Yaomin Hu

**Affiliations:** ^1^Department of Geriatrics, Ren Ji Hospital, Shanghai Jiao Tong University School of Medicine, Shanghai, China; ^2^Guangdong Institute of Intelligence Science and Technology, Hengqin, Zhuhai, China

**Keywords:** sarcopenia, nutrition indicators, AHLC, predicative model, sarcopenia risk

## Abstract

**Background:**

Sarcopenia, a condition characterized by low muscle mass, plays a critical role in the health of older adults. Early identification of individuals at risk is essential to prevent sarcopenia-related complications. This study aimed to develop a predictive model using readily available clinical nutrition indicators to facilitate early detection.

**Methods:**

A total of 1,002 participants were categorized into two groups: 819 with normal skeletal muscle mass (SMM) and 183 with low muscle mass (sarcopenia). A predictive model was developed for sarcopenia risk via multivariate logistic regression, and its performance was assessed using four analyses: receiver operating characteristic (ROC) curve analysis, decision curve analysis (DCA), a nomogram chart, and external validation. These methods were used to evaluate the model’s discriminative ability and clinical applicability.

**Results:**

In the low-SMM group, more females (55.73% vs. 40.42%) and older individuals (median 61 vs. 55 years) were observed. These patients had lower albumin (41.00 vs. 42.50 g/L) and lymphocyte levels (1.60 vs. 2.02 × 10^9^/L) but higher HDL (1.45 vs. 1.16 mmol/L) and calcium levels (2.24 vs. 2.20 mmol/L) (all *p* < 0.001). Using LASSO regression, we developed a nutritional AHLC (albumin + HDL cholesterol + lymphocytes + calcium) model for sarcopenia risk prediction. AUROC and DCA analyses, as well as nomogram charts and external validation, confirmed the robustness and clinical relevance of the AHLC model for predicting sarcopenia.

**Conclusion:**

Our study employs serum nutrition indicators to aid clinicians in promoting healthier aging. The AHLC model stands out for weight-independent evaluations. This novel approach could assess sarcopenia risk in the Chinese population, thereby enhancing aging and quality of life.

## Introduction

As the global aging issue intensifies, age-related health challenges like sarcopenia and malnutrition have increasingly attracted extensive attention in the field of gerontology (1). Sarcopenia is defined as a disease, the root causes of which include factors such as old age, decreased physical activity, insufficient nutrition, chronic diseases, malignant tumors and physical cachexia, leading to gradual weakening of skeletal muscle mass, strength and function (2–5). Malnutrition or undernutrition, on the other hand, could be defined as a condition in which changes in body composition (but not including loss of fat mass), a decrease in the number of body cells, resulting in a decline in physical and mental function, and a deterioration in clinical outcomes, primarily due to insufficient intake or absorption of nutrients (6).

Malnutrition is a common but underrecognized comorbidity among hospitalized older adults, and its prevalence typically varies between 30 and 55%, depending on the study population and the assessment tools used (7). Multiple studies have confirmed that malnutrition significantly increases the risk of sarcopenia in older adults. For example, a follow-up study conducted by Beaudart et al. found that older adults who were malnourished had a four-fold higher risk of developing sarcopenia (8), which indicates that sarcopenia is strongly associated with insufficient protein or nutrient intake (9). Previous study showed that insufficient protein intake destroys the balance of protein catabolism and stimulates the occurrence of skeletal muscle atrophy and impaired muscle growth, resulting in decreased muscle mass and physical function in the elderly (10).

Despite numerous studies suggesting a potential connection between malnutrition and sarcopenia (8, 9), this area remains relatively underexplored. Timely screening for sarcopenia through nutritional assessment and inclusion in personalized nutrition and exercise interventions could enhance management and improve physical and mental well-being, as well as quality of life (11).

Over the years, various predictive tools, such as the simple five-item questionnaire (SARC-F), have been developed to identify individuals at risk of sarcopenia (12). While tools like SARC-F provide valuable insights, they often rely on subjective measures that may not be universally accessible in resource-limited settings. Moreover, indices such as the Prognostic Nutrition Index (PNI) (13), Nutritional Risk Index (NRI) (14), Geriatric Nutritional Risk Index (GNRI) (15), Controlling Nutritional Status (CONUT) score (16), and Body Mass Index (BMI) (17) are commonly used for quantifying nutritional risk, but they lack specificity of nutritional indicators for sarcopenia diagnosis.

To address these gaps, this study aimed to conduct a cross-sectional investigation and develop a risk prediction model for sarcopenia diagnosis (low skeletal muscle mass) using objective clinical nutritional indicators in a Chinese population aged 18 years and older. The results were compared with established indices, including PNI, NRI, GNRI, CONUT score, and BMI. By leveraging readily accessible nutritional biomarkers, this study seeks to enhance the accuracy and applicability of sarcopenia risk prediction, providing robust theoretical backing and practical guidance for advancing healthy aging initiatives.

## Methods

### Study design and population

This retrospective cross-sectional study was conducted in the Department of Geriatrics, Ren Ji Hospital, School of Medicine, Shanghai Jiao Tong University from December 2020 to August 2023. In accordance with the Declaration of Helsinki, this study has been approved by the Ethics Committee of Ren Ji Hospital, School of Medicine, Shanghai Jiao Tong University (No. KY2021-071-B). Prior to inclusion in the study, all participants had signed informed consent for the use of their health examination data. Study participants were required to meet the following criteria: have undergone regular health checkups and bioelectrical impedance analysis (BIA), and be at least 18 years of age. Exclusion criteria include: (1) severe systemic disease, such as severe infection, malignancy or multiple organ failure (including liver, kidney, respiratory system or heart); (2) have been diagnosed with a neuromuscular disease; (3) have peripheral edema or have received diuretic treatment in the past month; (4) Oral, inhalation or nasal use of glucocorticoids; (5) Received nutritional support, such as gastric tube or nasal feeding enteral nutrition or peripheral deep vein parenteral nutrition; and (6) There is incomplete data. Additionally, a total of 460 participants were included for external validation. These participants were enrolled from Ren Ji Hospital, School of Medicine, Shanghai Jiao Tong University between September 2023 and June 2024.

### Data collection

After obtaining consent from the participants, trained staff measured their height and weight. We collected information about their medical history, including chronic conditions such as hypertension, type 2 diabetes, coronary heart disease, hyperlipidemia, cerebral infarction, as well as their history of alcohol and tobacco use using questionnaires.

All participants fasted for 8 h following their dinner on the evening prior to the physical examination. Fasting venous blood samples were collected and treated with heparin for anticoagulation. The collected blood samples were stored at a temperature of −20°C and sent to the laboratory at Ren Ji Hospital, School of Medicine, Shanghai Jiao Tong University for analysis. A wide range of biochemical markers were measured, including blood routine parameters (hemoglobin, lymphocyte count, C-reactive protein), blood biochemistry (albumin, aspartate aminotransferase, alanine aminotransferase, urea nitrogen, creatinine, uric acid, total cholesterol, triglycerides, low-density lipoprotein cholesterol, high-density lipoprotein cholesterol, fasting blood glucose, glycated hemoglobin, calcium, phosphorus, magnesium), serum ferritin, 25(OH) vitamin D, and thyroid function tests (FT3, FT4, TSH).

All these assays underwent rigorous quality control procedures, conducted by trained laboratory personnel, and were tested using Hitachi 7,600–110 and Hitachi 7,020 automatic analyzers (Hitachi, Tokyo, Japan). Reagents used for these assays were provided by the respective companies.

### Nutritional assessments

Contents and characteristics of the five nutritional indexes are shown in Supplementary Tables S1, S2. PNI is calculated as serum albumin (g/L) + 5 × total lymphocyte count (×10^9^/L) (13); NRI is calculated according to the following formula: NRI = (1.519 × serum albumin) (g/L) +41.7 × (present weight/ideal body weight), which is suitable for patients under 60 years old (14); GNRI is calculated as follows: GNRI = (1.489 × serum albumin) (g/L) + 41.7 × (present weight/ideal body weight), which is suitable for patients aged 60 years and above; The ideal weight is calculated using the Lorentz equation (15); CONUT score is calculated based on levels of serum albumin, total lymphocyte count and total cholesterol (16); BMI = weight (kg)/height^2^ (m^2^) (17).

### Muscle mass measurement

Limb skeletal muscle mass (SMM) was measured using Bioelectrical Impedance Analysis (BIA) with the InBody770 device (InBody, Seoul, Korea). Before the examination, participants were instructed to fast, empty their bladders, and rest for at least 15 min. Limb BIA measurement evaluates limb body composition by positioning four electrodes (two on the wrists and two on the ankles) on the participant’s limbs. We utilized the square of height to adjust for SMM and calculate the Limb Skeletal Muscle Mass Index (SMI), defined as SMM (Kg)/height (m^2^). According to the SMI value, women <5.7 kg/m^2^ and men <7.0 kg/m^2^ were diagnosed with sarcopenia (4).

### Statistical analysis

Continuous variables were expressed as medians (range), while categorical variables were expressed as the number of patients (percentage, %). The Wilcoxon test (Mann–Whitney U test) was employed to compare differences among continuous variables, and the χ2 test was used to assess differences among categorical variables. Subsequently, we conducted Pearson correlation analysis to explore the relationship between SMI and relevant variables. Lasso regression was applied for variable selection. Univariate and multivariate logistic regression analyses were performed to predict the potential risk of sarcopenia. We evaluated the predictive performance of different models using calibration curve analysis, AUROC analysis, and DCA analysis. In the AUROC analysis, we determined the optimal classification threshold by maximizing the Youden’s index (18), which effectively balances the sensitivity and specificity of the predictive model. Model calibration analysis was conducted to mitigate the impact of confounding factors. A nomogram was utilized to visualize the relationships between variables in the prediction model.

All statistical analyses were carried out using R software (version 4.2.3, https://www.r-project.org/). Statistical significance was considered when double-tailed *p* values were < 0.05.

## Results

### Basic characteristics of the study population

**Figure 1:**
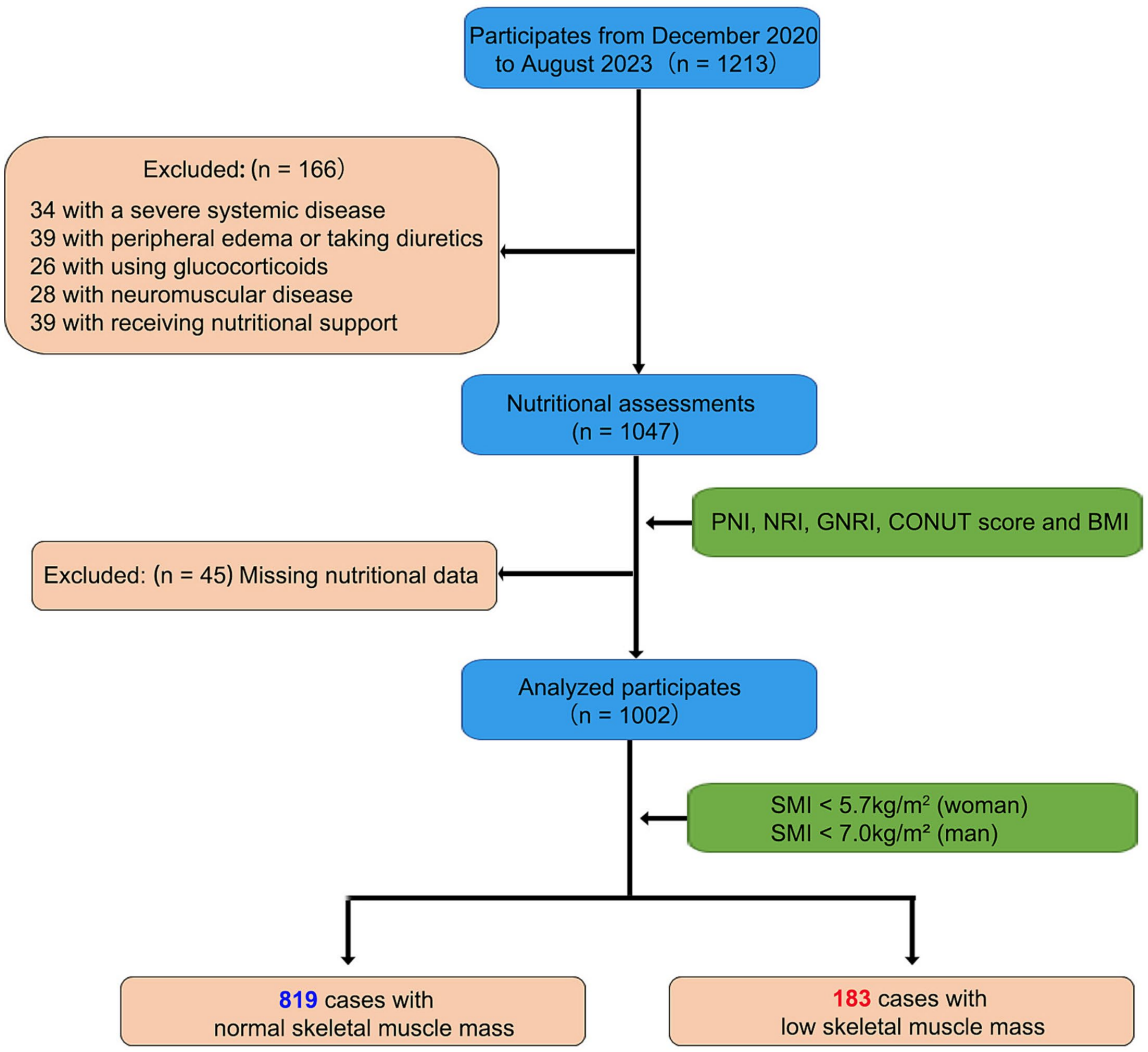
The flow diagram of study population. Initially, 1,213 subjects were enrolled from the Geriatric Department. Of these, 166 patients were excluded due to severe disease or medication issues, and 45 patients were excluded due to missing nutritional data. The final analysis included 1,002 eligible subjects: 819 with normal skeletal muscle mass and 183 with low skeletal muscle mass. This diagram provides an overview of the inclusion and exclusion criteria, detailing the final study sample size and data availability.

During the study period, a total of 1,213 subjects who visited the Geriatric Department, including individuals under 59 years of age receiving specialized care, were enrolled. Subsequently, 166 patients were excluded from the analysis, and an additional 45 patients were found to have missing nutritional data. Ultimately, our analysis focused on the data of 1,002 eligible patients (Figure 1). We measured SMI values via BIA and classified these patients according to the criteria proposed by the Asian Sarcopenia Working Group (AWGS) (19).

A total of 183 participants (18.3%) were classified as having low skeletal muscle mass (SMM), while 819 participants (81.7%) were classified as having normal SMM based on SMI values obtained via BIA (Table 1). Significant differences were observed between the low SMM and normal SMM groups in gender, age, BMI, and most nutrition-related biochemical markers (all *p* < 0.05). The low SMM group comprised a higher proportion of women, older individuals, and participants with lower BMI. Additionally, lymphocyte count, hemoglobin, albumin, triglyceride, ferritin, creatinine, and uric acid levels were significantly lower, while HDL and calcium levels were significantly higher in the low SMM group. Nutritional indices such as NRI and GNRI were also decreased, whereas PNI and CONUT scores were increased in the low SMM group. No significant differences were observed in AST, cholesterol, LDL, FT4, TSH, or phosphorus levels between the two groups (Table 1).

**Table 1:** Clinical characteristics of participants with normal and low skeletal muscle mass (SMM).

Characteristics	All subjects	Normal SMM	Low SMM (sarcopenia)
*N* (%)	1,002	819 (81.7%)	183 (18.3%)
Sex (%)			
Female	433 (43.21)	331 (40.42)	102 (55.74) ^***^
Male	569 (56.79)	488 (59.59)	81 (44.26) ^***^
Age stage (%)			
<60 (age)	636 (63.47)	557 (68.01)	79 (43.17) ^***^
[60, 80]	320 (31.94)	235 (28.69)	85 (46.45)
≥80 (age)	46 (4.59)	27 (3.30)	19 (10.38)
Age	56 [47, 65]	55 [47, 63]	61 [49, 72] ^***^
Height (m)	1.68 [1.60, 1.73]	1.69 [1.62, 1.74]	1.62 [1.57, 1.68] ^***^
BMI (Kg/m^2^)	23.50 [21.29, 25.85]	24.29 [22.30, 26.33]	19.86 [18.50, 21.02] ^***^
Weight (Kg)	65.95 [56.23, 74.98]	68.70 [60.30, 77.05]	51.50 [47.40, 57.45] ^***^
SMI (Kg/m^2^)	7.10 [6.20, 7.90]	7.40 [6.50, 8.10]	5.60 [5.30, 6.60] ^***^
Lymphocytes (×10^9^/L)	1.96 [1.58, 2.43]	2.02 [1.67, 2.48]	1.60 [1.32, 2.13] ^***^
Hemoglobin (g/L)	136.00 [125.00, 147.00]	138.00 [127.00, 148.00]	129.00 [120.00, 140.00] ^***^
ALT (U/L)	18.00 [13.00, 25.00]	18.00 [13.00, 26.00]	15.00 [12.00, 21.00] ^***^
AST (U/L)	18.00 [15.00, 22.00]	18.00 [15.00, 22.00]	19.00 [16.00, 22.00]
Albumin (g/L)	42.30 [40.30, 44.70]	42.50 [40.70, 44.85]	41.00 [38.15, 44.05] ^***^
Triglyceride (mmol/L)	1.30 [0.92, 1.86]	1.35 [0.99, 1.96]	1.01 [0.68, 1.43] ^***^
Cholesterol (mmol/L)	185.76 [162.15, 211.30]	185.76 [161.38, 211.30]	185.37 [165.06, 216.14]
HDL (mmol/L)	1.19 [1.00, 1.46]	1.16 [0.97, 1.38]	1.45 [1.18, 1.76] ^***^
LDL (mmol/L)	2.82 [2.29, 3.40]	2.83 [2.30, 3.43]	2.74 [2.23, 3.26]
Creatinine (mmol/L)	61.00 [50.00, 72.00]	62.00 [51.00, 72.00]	57.00 [46.50, 69.00] ^***^
Urea nitrogen (μmol/L)	5.10 [4.40, 5.90]	5.00 [4.30, 5.80]	5.40 [4.40, 6.30] ^*^
Uric acid (μmol/L)	325.00 [269.25, 384.00]	335.00 [278.50, 391.00]	293.00 [245.00, 348.00] ^***^
FT3 (pmol/L)	4.67 [4.31, 5.11]	4.72 [4.34, 5.14]	4.56 [4.14, 4.89] ^***^
FT4 (pmol/L)	15.90 [14.63, 17.40]	15.90 [14.70, 17.40]	15.80 [14.40, 17.90]
TSH (mIU/L)	2.09 [1.43, 3.02]	2.10 [1.450 3.02]	2.08 [1.330, 3.00]
Calcium (mmol/L)	2.21 [2.14, 2.28]	2.20 [2.14, 2.27]	2.24 [2.16, 2.32] ^***^
Phosphorus (mmol/L)	1.15 [1.05, 1.26]	1.15 [1.06, 1.26]	1.16 [1.05, 1.31]
Magnesium (mmol/L)	0.91 [0.87, 0.95]	0.91 [0.87, 0.95]	0.93 [0.88, 0.97] ^*^
CRP (mg/l)	0.67 [0.37, 1.42]	0.77 [0.38, 1.44]	0.47 [0.35, 1.26] ^***^
Fasting blood sugar (mmol/L)	4.70 [4.20, 5.39]	4.68 [4.19, 5.34]	4.83 [4.25, 5.50]
Glycated hemoglobin (%)	5.60 [5.30, 6.00]	5.60 [5.30, 6.00]	5.50 [5.20, 5.80] ^*^
Serum total 25 (OH)D (ng/ml)	19.99 [16.27, 23.96]	20.08 [16.43, 23.81]	18.58 [15.28, 24.67]
Ferritin (μg/L)	112.45 [58.40, 210.23]	118.70 [59.45, 215.90]	93.20 [54.45, 161.20] ^*^
NRI	109.25 [103.23, 115.29]	111.40 [105.53, 116.58]	100.39 [95.53, 104.79] ^***^
GNRI	107.99 [102.02, 113.92]	110.12 [104.28, 115.30]	99.10 [94.34, 103.55] ^***^
PNI	52.70 [49.51, 55.59]	53.25 [50.25, 56.00]	50.00 [46.23, 52.93] ^***^
CONUT	1 [0, 1]	1 [0, 1]	1 [0, 2] ^***^

Data presented as median (interquartile range, IQR). Statistical significance determined by *p* < 0.05. *, *p* < 0.05; ***, *p* < 0.001. BMI, Body Mass Index; SMI, Muscle Mass Index; ALT, alanine transaminase; AST, aspartate aminotransferase; HDL, high-density lipoprotein cholesterol; LDL, low-density lipoprotein cholesterol; FT3, free Triiodothyronine; FT4, free Thyroxine; TSH, thyroid-stimulating hormone; CRP, C-reactive protein; NRI, nutritional risk index; GNRI, geriatric nutritional risk index; PNI, prognostic nutritional index; CONUT, controlling nutritional status.

The Pearson correlation analysis indicated strong positive correlations (R > 0.6, *p* < 0.001) between SMI and weight-related parameters, including weight (R = 0.89), height (R = 0.73), BMI (R = 0.73), NRI (R = 0.62), GNRI (R = 0.62), PNI (R = 0.33). In terms of clinical biochemical markers, SMI demonstrated significant positive correlations (R ≥ 0.3, *p* < 0.001) with hemoglobin (R = 0.57), albumin (R = 0.30), uric acid (R = 0.52), creatinine (R = 0.50), ferritin (R = 0.41) and triglyceride (R = 0.37), while it exhibits a significant negative correlation (R = −0.47, *p* < 0.001) with HDL. Other correlations with different indicators appear comparatively weaker (all absolute R < 0.30) (Supplementary Figure S1).

### Development and comparative performance of a predictive model for sarcopenia

We initially attempted to construct a new assessment model by incorporating variables that exhibited significant differences in baseline comparisons and demonstrated significant associations with SMI values in Lasso regression analyses. However, when weight-related indicators were present, the importance of weight and gender variables overshadowed that of other indicators (e.g., lambda decreased, the coefficients of weight and sex increased, while the coefficients of other indicators approached 0) (Supplementary Figure S2A). This made it challenging to discern the relative importance of non-weight-related measures.

To address this issue, we performed a modified Lasso regression analysis, excluding weight-related variables. In the weight-independent model (Supplementary Figure S2B), when lambda is relatively low at exp.(−6), the absolute values of the coefficients for the Albumin, Age stage, Calcium, HDL, and Lymphocytes indicators rank among the top five, with values of (−0.748, 0.741, 0.654, 0.567, −0.442), respectively. As lambda increases to exp.(−3), only these five variables still have significant coefficients (HDL = 0.411, Age stage = 0.254, Albumin = −0.178, Lymphocytes = −0.140, Calcium = 0.085), while the others are excluded (Supplementary Figure S2B). Consequently, we identified these five prominent variables subsequent modeling, aiming to distinguish between the two population groups.

Following variable selection process, we proceeded with additional univariate and multivariate Logistic regression analyses (Supplementary Table S3) and constructed several models. The model related to weight was named WS: Weight + Sex. In contrast, models independent of body weight were named as AHL: Albumin + HDL + Lymphocytes, AHLC: Albumin + HDL + Lymphocytes + Calcium, and AHLCA: Albumin + HDL + Lymphocytes + Calcium + Age stage. The results demonstrated that the selected variables (Weight, Sex, Age stage, Calcium, HDL, Lymphocytes, and Albumin) were all statistically significant in univariate Logistic regression analysis (all *p* < 0.001). Additionally, models WS (AUC = 0.935), AHL (AUC = 0.780), AHLC (AUC = 0.805), and AHLCA (AUC = 0.812) constructed using these variables, all exhibited strong predictive performance (AUC ≥ 0.78, all *p* < 0.01).

To assess the robustness of these four models’ predictive capabilities, we conducted calibration curve analysis. After 1,000 bootstrap replicates, the original and calibrated prediction curves for these four models including WS (mean absolute error = 0.006), AHLC (mean absolute error = 0.011), AHL (mean absolute error = 0.009) and AHLCA (mean absolute error = 0.009) closely matched the ideal curve (Supplementary Figures S2C–F).

### The combination of AUROC and DCA analysis confirms the robustness and clinical relevance of the AHLC model

To comprehensively assess the performance of our newly developed models, we conducted a series of comparisons, including established indices such as NRI, GNRI, BMI, PNI, and CONUT score, which are currently recognized to quantify nutritional risk. Additionally, we included univariate models associated with these parameters for validation and comparison. We randomly divided our dataset and employed 10-fold cross-validation with 10 repetitions for both validation (Supplementary Table S4) and training set.

The results of validation set revealed that the WS model (AUC = 0.934, Accuracy = 0.877 and Youden index = 0.773) exhibited the highest performance, which was superior to the univariate model focused solely on weight (AUC = 0.885, Accuracy = 0.800 and Youden index = 0.654) (Supplementary Table S4). In the category of models independent of weight, both AHLCA (AUC = 0.806, Accuracy = 0.758 and Youden index = 0.547) and AHLC (AUC = 0.802, Accuracy = 0.781 and Youden index = 0.544) exhibited robust performance, with minimal performance differences between them (Supplementary Table S4).

**Figure 2:**
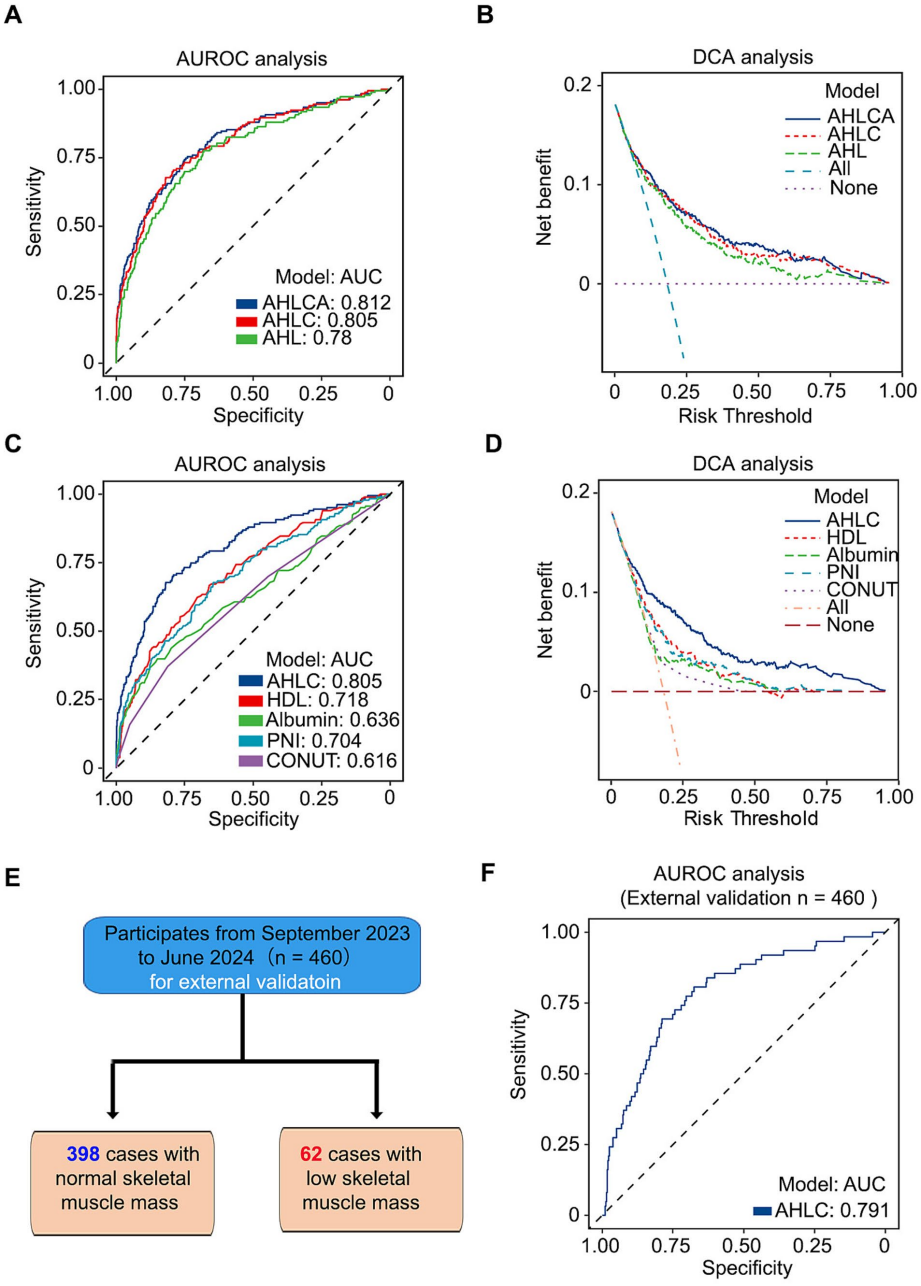
The combination of AUROC and DCA analysis confirms the robustness and clinical relevance of the AHLC model. **(A)** AUROC analysis of three weight-independent models, illustrating their predictive performance. **(B)** DCA analysis of the same three models, highlighting their clinical utility. **(C)** AUROC analysis comparing the AHLC model with other weight-independent models. **(D)** DCA analysis comparing the AHLC model with other weight-independent models, assessing clinical relevance. **(E)** Flow diagram of the study population for external validation, detailing the enrollment and analysis process with 460 subjects, including 398 with normal skeletal muscle mass and 62 with low skeletal muscle mass. **(F)** AUROC analysis of the AHLC model in the external validation dataset, confirming its predictive accuracy in a different cohort. AUROC (Area Under the Receiver Operating Characteristic Curve) and DCA (Decision Curve Analysis) values are used to assess model performance and clinical utility. AUROC, area under the receiver operating characteristic curve; DCA, decision curve analysis; AHLC, Albumin + HDL + Lymphocytes + Calcium.

Furthermore, we carried out an analysis of the area under the receiver operating characteristic curve (AUROC) and decision curve analysis (DCA) to compare the effectiveness of different models. Among models independent of weight, AHLCA (AUC = 0.812) and AHLC (AUC = 0.805) emerged as top performers (Figures 2A–D). In accordance with the Occam’s razor principle (20), when model performance was comparable, we choose the AHLC model, characterized by fewer variables.

To further evaluate the discriminative ability and clinical applicability, we conducted external validation with a total of 460 participants enrolled from Ren Ji Hospital, School of Medicine, Shanghai Jiao Tong University, Shanghai, China, the same hospital where the primary study population was recruited (Figure 2E; Supplementary Table S5). The AUC for the external validation of the AHLC model was 0.791 (Figure 2E; Table 2). Overall, both internal and external validation (Table 2) demonstrated that the AHLC model has strong screening ability, indicating its potential clinical application value.

**Table 2:** Validation of the AHLC model through internal and external validation methods in the study population.

Models	AUC	Sensitivity	Specificity	Accuracy	Positive predicting value	Negative predicting value	Positive likelihood ratio	Negative likelihood ratio	Youden Index
AHLC (Training set, *n* = 902)	0.805	0.699	0.800	0.782	0.440	0.923	3.531	0.376	0.499
AHLC (Internal validation set, *n* = 100)	0.802	0.759	0.786	0.781	0.469	0.938	4.354	0.299	0.544
AHLC (External validation set, *n* = 460)	0.791	0.694	0.789	0.776	0.339	0.943	3.286	0.388	0.482

Data presented as individual values of statistical metrics. AUROC (area under the receiver operating characteristic curve), DCA (decision curve analysis); AHL (Albumin + HDL + Lymphocytes); AHLC (Albumin + HDL + Lymphocytes + Calcium); AHLCA (Albumin + HDL + Lymphocytes + Calcium + Age Stage); PNI (prognostic nutritional index); RNI (nutritional risk index); GNRI (geriatric nutritional risk index); CONUT (controlling nutritional status); BMI (Body Mass Index); AHLC (Albumin + HDL + Lymphocytes + Calcium).

After finalizing the model, we conducted variable adjustment to alleviate the potential influence of confounding variables. The results emphasized that AHLC model maintained significant predictive capability (all *p* < 0.001) even after variable adjustment (Supplementary Table S6).

In summary, when considering predictors associated with weight or weight-related indicators, the WS model proves to be the most appropriate choice for predicting muscle mass deficiency. Conversely, when utilizing measures unrelated to body weight for predictions, the AHLC model stands out as the optimal choice.

### Nomograms for sarcopenia using AHLC score

**Figure 3:**
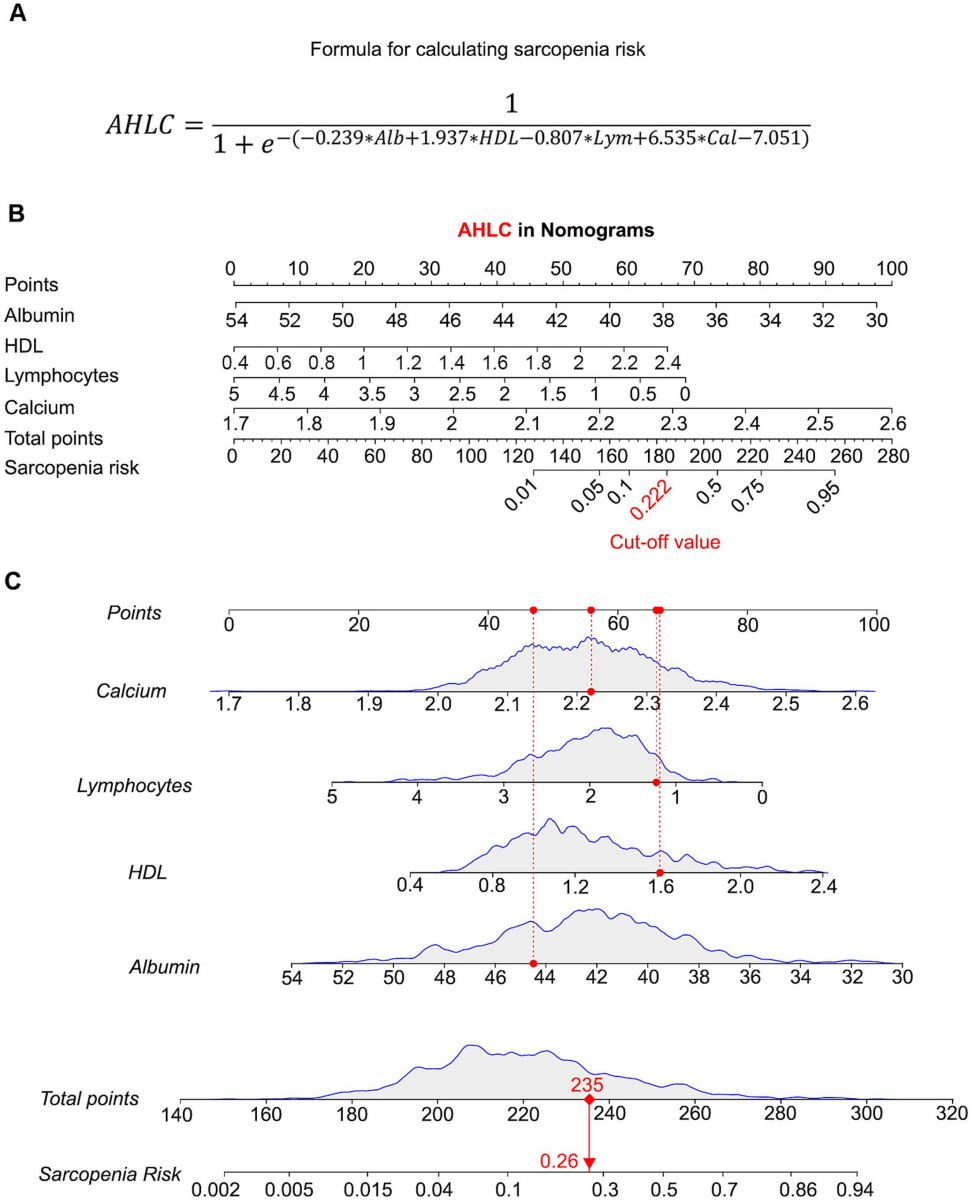
Predictive validity of AHLC models using nomograms. **(A)** A formula for sarcopenia risk calculation of AHLC model. **(B)** Nomogram for the AHLC model, showing how each variable contributes to the total risk score. The corresponding risk value cut-off for the AHLC model, set at 0.222, used to classify the risk of sarcopenia. Nomograms are used to visualize the predictive power of the AHLC model, with the cut-off value indicating the threshold for risk classification. **(C)** Individual Risk Evaluation with the AHLC nomogram for Sarcopenia Assessment. Each line represents an individual biological indicator. The curve above each line shows the statistical distribution of that indicator, with the red dot indicating the specific measurement value for an example individual. The red dashed line shows the position of this value within the distribution. The total score is calculated based on these indicators, which is used to estimate the risk of sarcopenia. Calcium: Red dot corresponds to 2.22 mmol/L. Lymphocytes: Red dot corresponds to 1.23 × 10^9/L. HDL: Red dot corresponds to 1.61 mmol/L. Albumin: Red dot corresponds to 44.5 g/L. Total points: Sum of the weighted scores for each biological indicator, resulting in a total score of 235 points. The sarcopenia risk, estimated based on the total score, is indicated by the red dot on the risk curve, which shows a risk value of 0.26. The red arrow connects the total score to the corresponding sarcopenia risk on the curve, indicating the risk probability. AHLC, Albumin + HDL + Lymphocytes + Calcium; HDL, high-density lipoprotein.

To visualize AHLC models, we generated a nomogram using an explicit formula (Figure 3A) that provides a graphical representation. Each variable corresponds to a specific value and is assigned an associated score, initially located in the first row (Figure 3B). Subsequently, by summing the scores for each variable and identifying the total score on the respective scale, the nomogram facilitates the calculation of the probability of sarcopenia development. In the AHLC model (with a sensitivity of 0.759 and specificity of 0.786, as shown in Table 2), it signifies a higher predicted risk of sarcopenia for the individual. When the risk value, which is precisely calculated by the formula, surpasses 0.222 in the AHLC model (Figure 3C).

## Discussion

Sarcopenia is a common health problem in the elderly, which seriously affects the quality of life and overall health status (21). In this study, we introduced the AHLC model, which integrates albumin, HDL cholesterol, lymphocytes, and calcium as key predictors of sarcopenia risk. Our results confirmed the model’s accuracy and clinical utility, as validated by AUROC, DCA, and nomogram analyses. This novel approach offers a simple, nutrition-based method for identifying individuals at risk of sarcopenia, providing an accessible and practical tool for early intervention and promoting healthy aging, especially in the Chinese adult’s population.

### Construction of weight-related WS model and the weight-independent AHLC model

Previous studies have consistently demonstrated a strong association between malnutrition and sarcopenia (22). In a two-center prospective cohort study that included 350 hospitalized older adults (mean age: 77.2 ± 7.6 years, 56% of whom were women), 98 participants (28%) tragically died during 2 years of follow-up. The study found that participants with malnutrition-sarcopenia syndrome had the highest risk ratio (HR: 19.8) (23). Additionally, the EFFORT study highlighted the significance of early intensive nutritional therapy, reducing mortality by 35% and improving clinical outcomes, including a 21% reduction in intensive care unit admissions, major complications, readmissions, and decreased function (24). These findings highlight the pivotal role of nutrition in the development of sarcopenia and provide important background support for our study.

In this study, we conducted a cross-sectional analysis involving 1,002 participants who underwent health checkups and bioelectrical impedance analysis (BIA). Results indicated that 18.3% of the participants were classified as having low skeletal muscle mass (sarcopenia). Statistically significant differences in nutritional status (e.g., NRI, GNRI, PNI, CONUT score, BMI, etc.) were observed between individuals with low skeletal muscle mass and those with normal skeletal muscle mass (*p* < 0.001). Furthermore, individuals with low skeletal muscle mass were predominantly female and of older age, consistent with previous research findings (1, 25, 26).

In the construction of potential sarcopenia risk prediction models, we initially employed Lasso regression analysis for variable selection. Notably, weight and gender exhibited such high importance that other indicators were overshadowed. Consequently, we developed two distinct models: the weight-related WS model and the weight-independent AHLC model. Both models demonstrated strong performance in predicting sarcopenia risk and provided clinicians with valuable assessment tools in different clinical contexts. When compared to widely recognized quantitative nutritional risk indicators (e.g., NRI, GNRI, PNI, CONUT score, BMI), the WS model performed the best (AUC = 0.935) among weight-related models (NRI, GNRI, BMI, WS), while among weight-independent models (PNI, CONUT score, AHLC), the AHLC model exhibited the most favorable performance (AUC = 0.805).

Our findings consistently highlight the critical roles of weight and gender in predicting the risk of sarcopenia, which is consistent with previous findings (27–29). Importantly, it is worth noting that among individuals with sarcopenia, there are those with sarcopenic obesity (SO) (30), and SO is associated with multiple adverse health outcomes, including frailty, falls, disability, increased morbidity, and mortality (31). In such cases, body weight alone does not serve as an independent predictor.

### Clinical serum nutritional AHLC model in predicting sarcopenia risk

Thus, we further developed the clinical serum nutritional AHLC model to provide a more objective indicator of potential sarcopenia risk in the absence of weight effects. This model incorporates four serological indicators: albumin, high-density lipoprotein (HDL), blood calcium, and lymphocyte counts, providing a comprehensive assessment of nutritional status, lipid metabolism, skeletal muscle health, and immune function—factors that play pivotal roles in the onset and progression of sarcopenia (21).

Secondary data analysis from our China Health and Retirement Longitudinal Study indicated that higher serum triglyceride to HDL cholesterol ratios in elderly diabetic patients were associated with better muscle status (32). Furthermore, mounting evidence suggests a high co-occurrence rate of sarcopenia and osteoporosis (33), with shared biological pathways including aging, inflammation, hormonal imbalances, and nutritional deficiencies (34–37). Consequently, some researchers advocate for considering osteoporosis and sarcopenia as a unified entity: osteosarcopenia (38–40). This revelation underscores the importance of addressing bone and muscle health in an integrated manner when managing the health of the elderly, rather than addressing these issues in isolation.

To facilitate comprehension of sarcopenia risk among clinicians, caregivers, and older adults, we developed a nomogram for predicting the potential risk of sarcopenia. This nomogram can visually show the individual’s risk value (41). When the Value at Risk (VAR) of the AHLC score exceeds 0.222, an individual may face a heightened risk of potential sarcopenia. This tool holds promise for the early identification of sarcopenic individuals, allowing for interventions to enhance quality of life and prevent the onset of decreased muscle function and related complications.

Nevertheless, it is essential to acknowledge the limitations of this study. Firstly, the relatively small sample size may introduce selective bias. We were only able to gather data on muscle mass, and unfortunately, we lacked information on muscle strength or functionality. Future research should encompass larger, multi-center studies to validate and refine the application of AHLC scoring. Another potential limitation of our study is the single-center design, which may limit the generalizability of the AHLC model to other populations. However, recent evidence suggests a strong association between elevated HDL-C levels and an increased risk of sarcopenia and reduced grip strength in older adults (42). These findings align with our model, where HDL-C emerged as a significant predictor of sarcopenia risk, reinforcing the biological relevance of this marker. To further validate the AHLC model and ensure its applicability across diverse populations, future studies will leverage external datasets from varied regions and demographic groups. This approach will help establish the model’s robustness and expand its utility in global clinical settings. In addition, while acute inflammatory conditions were accounted for using CRP levels, chronic inflammatory diseases or long-term medication use may also influence biomarkers such as lymphocytes and albumin. Future studies should incorporate additional adjustments or sensitivity analyses to account for these potential confounders.

While the biomarkers used in our model (such as albumin and lymphocytes) are commonly available in hospital settings, we acknowledge that they may not be as easily accessible in low-resource environments. In such cases, other simple and widely used methods for diagnosing sarcopenia, such as grip strength and calf circumference, could be incorporated into the model as alternatives. In future studies, we plan to explore how the model can be applied in these settings, and whether including additional simple measures like grip strength and calf circumference would enhance the model’s effectiveness in low-resource environments. Additionally, our current studies are focusing on interventions and treatments for sarcopenia. Early-stage tools for identifying sarcopenia risk are crucial for selecting appropriate interventions. By improving nutrition, increasing exercise, and maintaining a healthy weight, we could help older adults reduce their risk of sarcopenia and maintain optimal muscle mass and function (43, 44). Achieving a healthier and more vibrant aging society necessitates collaboration among the healthcare sector, healthcare institutions, and the community to collectively enhance the overall health of older individuals.

## Data Availability

The original contributions presented in the study are included in the article/Supplementary material, further inquiries can be directed to the corresponding authors.

## Glossary

SMM: skeletal muscle mass
BIA: bioelectrical impedance analysis
ROC: receiver operating characteristic
DCA: decision curve analysis
BMI: body mass index
SMI: muscle mass index
ALT: alanine transaminase
AST: aspartate aminotransferase
HDL: high-density lipoprotein cholesterol
LDL: low-density lipoprotein cholesterol
FT3: free triiodothyronine
FT4: free thyroxine
TSH: thyroid-stimulating hormone
CRP: C-reactive protein
NRI: nutritional risk index
GNRI: geriatric nutritional risk index
PNI: prognostic nutritional index
CONUT: controlling nutritional status
AHL: Albumin + HDL + Lymphocytes
AHLC: Albumin + HDL + Lymphocytes + Calcium
AHLCA: Albumin + HDL + Lymphocytes + Calcium + Age
WS: Weight + Sex
