# Supplementary material for: Development and validation of a predicative model for identifying sarcopenia in Chinese adults using nutrition indicators (AHLC)
Source: Front Nutr. 2024 Dec 12;11:1505655. doi: 10.3389/fnut.2024.1505655 (PMC11670750; doi:10.3389/fnut.2024.1505655)
Supplement: Supplementary file 1 [file Table_1.pdf]

Supplementary Table 1 Assessment indexes and approaches of nutritional indexes

Tools	Year of validation	Characteristics	Applied parameters	Assessment approach	
				Assessment outcomes	Malnutrition risk
PNI	1984	Quantifying the nutritional and immunological status.	PNI = serum albumin (g/L) + 5 × total lymphocyte count (×10^9/L)	>55<br>50-55<br>45-50<br><45	Normal<br>Light risk<br>Moderate risk<br>Severe risk
NRI	1980	To detect malnutrition and its associations to postoperative complications	NRI = (1.519 ×serum albumin)(g/L) + 41.7 ×(present weight/ideal body weight)	>100<br>97.5-100<br>83.5-97.5<br><83.5	Normal<br>Light risk<br>Moderate risk<br>Severe risk
GNRI	2005	Quantitative assessment of malnutrition risk especially designed for the elderly	GNRI = (1.489×serum albumin) (g/L) + 41.7 ×(present weight/ideal body weight)	>98<br>92-98<br>82-92<br>< 82	Normal<br>Light risk<br>Moderate risk<br>Severe risk
CONUT score	2005	A screening tool for early detection of poor nutritional status	Serum albumin, total lymphocyte count, and total cholesterol	0-1<br>2-4<br>5-8<br>9-12	Normal<br>Light risk<br>Moderate risk<br>Severe risk
ESPEN 2015	2015	Diagnosis consensus of malnutrition independent of clinical setting and etiology	BMI<20 kg/m^2（<70 years）or <22 kg/m^2（≥70 years）<br>BMI<18.5 kg/m^2（<70 years）or <20 kg/m^2（≥ 70 years）	BMI cut-off values	moderate malnutrition<br>severe malnutrition

PNI: Prognostic Nutritional Index; RNI: Nutritional Risk Index; GNRI: Geriatric Nutritional Risk Index; CONUT: Controlling Nutritional Status; Ideal body weight was calculated based on Lorentz formula: Ideal body weight (men) = height − 100 − ((height − 150)/4), Ideal body weight (women) = height − 100 − [(height − 150)/2]; ESPEN: European Society of Clinical Nutrition and Metabolism; BMI：Body Mass Index ，

BMI = weight (kg) / height^2 (m^2
