# Supplementary material for: Development and validation of a predicative model for identifying sarcopenia in Chinese adults using nutrition indicators (AHLC)
Source: Front Nutr. 2024 Dec 12;11:1505655. doi: 10.3389/fnut.2024.1505655 (PMC11670750; doi:10.3389/fnut.2024.1505655)
Supplement: Supplementary file 2 [file Table_2.pdf]

Supplementary Table 2 Controlling nutritional status (CONUT) score: assessment of malnutritional state

Parameter	malnutritional state			
	Normal	Mild	Moderate	Severe
Albumin (g/dL)[score]	≥3.50 [0]	3.00-3.49[2]	2.50-2.99[4]	<2.50[6]
Total lymphocyte count (mg/dL)[score]	≥1,600 [0]	1,200-1,599[1]	800-1,199[2]	<800 [3]
Total cholesterol (mg/dL)[score]	≥180 [0]	140-179[1]	100-139[2]	<100 [3]
Total score	0-1	2-4	5-8	9-12
