# Supplementary material for: Development and validation of a predicative model for identifying sarcopenia in Chinese adults using nutrition indicators (AHLC)
Source: Front Nutr. 2024 Dec 12;11:1505655. doi: 10.3389/fnut.2024.1505655 (PMC11670750; doi:10.3389/fnut.2024.1505655)
Supplement: Supplementary file 3 [file Table_3.pdf]

Supplementary table 3 Univariate and multivariate analysis on the different models

	Univariate analysis			Multivariate analysis with all Variables AHLCA (AUC = 0.812)		Multivariate analysis with all Variables AHLC (AUC = 0.805)		Multivariate analysis with all Variables AHL (AUC = 0.780)	
**Terms**	AUC	OR	CI	OR	CI	OR	CI	OR	CI
**Weight**	0.89	0.83***	0.81, 0.85						
**Sex**	0.58								
**Male**		0.54***	0.39, 0.74						
**Albumin**	0.64	0.84***	0.80, 0.89	0.80***	0.75, 0.85	0.79	0.74, 0.84***	0.86	0.81, 0.90
**HDL**	0.72	10.40***	6.49, 17	7.00***	4.13, 12.05	6.94	4.11, 11.88***	8.65	5.24, 14.50
**Lymphocytes**	0.68	0.34***	0.25, 0.46	0.50***	0.36, 0.70	0.45	0.32, 0.62***	0.46	0.33, 0.62
**Calcium**	0.60	36.86***	7.62, 182.12	499.11***	66.83, 4016.18	688.39	92.70, 5513.43***		
**Age stage**	0.63								
[60,80)		2.55***	1.81, 3.60	1.72**	1.16, 2.54				
[80,Inf)		4.96***	2.60, 9.30	2.99**	1.39, 6.33				

AHL (Albumin + HDL + Lymphocytes), AHLC (Albumin + HDL + Lymphocytes + Calcium), AHLCA (Albumin + HDL + Lymphocytes + Calcium + Age Stage), HDL (High-Density Lipoprotein).

*, P < 0.05; **, P< 0.01, ***, P < 0.001.
