# Supplementary material for: Development and validation of a predicative model for identifying sarcopenia in Chinese adults using nutrition indicators (AHLC)
Source: Front Nutr. 2024 Dec 12;11:1505655. doi: 10.3389/fnut.2024.1505655 (PMC11670750; doi:10.3389/fnut.2024.1505655)
Supplement: Supplementary file 4 [file Table_4.pdf]

**Supplementary Table 4 Comparison of training set metrics for different models**

| Models | AUC | Sensitivity | Specificity | Accuracy | Positive predicting value | Negative predicting value | Positive likelihood ratio | Negative likelihood ratio | Youden Index |
|---|---|---|---|---|---|---|---|---|---|
| NRI | 0.854 | 0.743 | 0.797 | 0.787 | 0.452 | 0.933 | 3.706 | 0.322 | 0.540 |
| GNRI | 0.855 | 0.745 | 0.797 | 0.787 | 0.452 | 0.933 | 3.713 | 0.320 | 0.541 |
| Weight | 0.885 | 0.857 | 0.739 | 0.761 | 0.424 | 0.959 | 3.294 | 0.193 | 0.596 |
| BMI | 0.896 | 0.792 | 0.882 | 0.865 | 0.599 | 0.950 | 6.703 | 0.236 | 0.674 |
| **AHL** | 0.781 | 0.755 | 0.702 | 0.712 | 0.364 | 0.928 | 2.569 | 0.347 | 0.457 |
| **AHLC** | 0.805 | 0.699 | 0.800 | 0.782 | 0.440 | 0.923 | 3.531 | 0.376 | 0.499 |
| **AHLCA** | 0.813 | 0.748 | 0.750 | 0.749 | 0.403 | 0.930 | 3.032 | 0.335 | 0.498 |
| PNI | 0.704 | 0.676 | 0.647 | 0.653 | 0.300 | 0.900 | 1.920 | 0.500 | 0.324 |
| CONUT | 0.616 | 0.371 | 0.812 | 0.731 | 0.306 | 0.853 | 1.975 | 0.774 | 0.183 |
| Albumin | 0.636 | 0.395 | 0.855 | 0.771 | 0.378 | 0.863 | 2.726 | 0.708 | 0.249 |
| HDL | 0.718 | 0.645 | 0.685 | 0.678 | 0.315 | 0.896 | 2.061 | 0.518 | 0.330 |

Modeling set for 10 repetitions of 10-fold cross-validation.
