# Supplementary material for: Development and validation of a predicative model for identifying sarcopenia in Chinese adults using nutrition indicators (AHLC)
Source: Front Nutr. 2024 Dec 12;11:1505655. doi: 10.3389/fnut.2024.1505655 (PMC11670750; doi:10.3389/fnut.2024.1505655)
Supplement: Supplementary file 5 [file Table_5.pdf]

Supplementary Table 5 Clinical characteristics between normal SMM and low SMM for external validation

**Characteristics**		**All subjects**	**Normal SMM**	**Low SMM**	**P value**	**Signfica nce**
n		460	398	62		
Sex (%)	female	191 (41.522)	153 (38.442)	38 (61.290)	0.001	***
	male	269 (58.478)	245 (61.558)	24 (38.710)		
SMI (Kg/m^2)		7.300 [6.300, 8.000]	7.400 [6.600, 8.100]	5.600 [5.325, 6.675]	<0.001	***
Weight (Kg)		68.050 [58.675, 76.825]	70.400 [61.525, 78.425]	52.400 [47.475, 57.775]	<0.001	***
Albumin (g/L)		42.300 [40.400, 44.725]	42.450 [40.700, 44.800]	40.700 [38.500, 44.425]	0.001	***
HDL (mmol/L)		1.170 [0.960, 1.422]	1.140 [0.940, 1.368]	1.375 [1.172, 1.760]	<0.001	***
Lymphocytes (x10^9/L])		2.070 [1.660, 2.530]	2.100 [1.690, 2.558]	1.785 [1.473, 2.320]	0.001	***
Calcium (median [mmol/L])		2.230 [2.170, 2.300]	2.230 [2.170, 2.300]	2.220 [2.172, 2.280]	0.928	
Age		54.000 [46.000, 61.000]	53.000 [46.000, 60.000]	60.000 [48.250, 69.000]	0.003	**
Age stage (%)	[0,60)	318 (69.130)	288 (72.362)	30 (48.387)	<0.001	***
	[60,80)	133 (28.913)	106 (26.633)	27 (43.548)		
	[80,Inf)	9 (1.957)	4 (1.005)	5 (8.065)		

BMI, Body Mass Index; SMI, Muscle Mass Index; ALT, alanine transaminase; AST, aspartate aminotransferase; HDL, high-density lipoprotein cholesterol; LDL, low-density lipoprotein cholesterol; FT3,free Triiodothyronine; FT4, free Thyroxine; TSH, thyroid-stimulating hormone; CRP, C-reactive protein; NRI nutritional risk index; GNRI geriatric nutritional risk index; PNI prognostic nutritional index; CONUT controlling nutritional status.
