# Supplementary material for: Development and validation of a predicative model for identifying sarcopenia in Chinese adults using nutrition indicators (AHLC)
Source: Front Nutr. 2024 Dec 12;11:1505655. doi: 10.3389/fnut.2024.1505655 (PMC11670750; doi:10.3389/fnut.2024.1505655)
Supplement: Supplementary file 6 [file Table_6.pdf]

Supplementary Table 6 Variable adjustment analysis AHLC model

Variables	AHLC			AHLC1			AHLC2			AHLC3		
	Crude OR	Crude CI	Crude p	Adjusted OR	Adjusted CI	Adjusted p	Adjusted OR	Adjusted CI	Adjusted p	Adjusted OR	Adjusted CI	Adjusted p
Albumin	0.788	0.739, 0.836	<0.001	0.795	0.746, 0.845	<0.001	0.810	0.755, 0.866	<0.001	0.789	0.733, 0.847	<0.001
HDL	6.937	4.11, 11.881	<0.001	7.065	4.056, 12.493	<0.001	6.162	3.196, 12.051	<0.001	5.975	1.868, 19.356	0.003
Lymphocytes	0.446	0.318, 0.616	<0.001	0.478	0.338, 0.665	<0.001	0.554	0.389, 0.777	0.001	0.535	0.371, 0.758	0.001
Calcium	688.391	92.699, 5513.425	<0.001	575.410	76.83, 4645.621	<0.001	1002.728	121.58, 9014.23	<0.001	920.098	100.984, 9102.369	<0.001

Lymphocytes, Hemoglobin, ALT, Albumin, Triglyceride, HDL, Uric acid, Calcium, LDL, FT4, Urea nitrogen and Creatinine; WS3: adjusted for Age, Lymphocytes, Hemoglobin, ALT, AST, Albumin, Triglyceride, Cholesterol, HDL, LDL, Creatinine, Urea nitrogen, Uric acid, FT3, FT4, TSH, Calcium, Phosphorus and Magnesium. AHLC: unadjusted model; AHLC1: adjusted for Age and Sex; AHLC2: adjusted for Age, Sex, Hemoglobin, ALT, Triglyceride, Uric acid, LDL, FT4, Urea nitrogen and Creatinine; AHLC3: adjusted for Age, Sex, Hemoglobin, ALT, AST, Triglyceride, Cholesterol, LDL, Creatinine, Urea nitrogen, Uric acid, FT3, FT4, TSH, Phosphorus, Magnesium and Magnesiu.
