# Supplementary material for: Development and validation of a predicative model for identifying sarcopenia in Chinese adults using nutrition indicators (AHLC)
Source: Front Nutr. 2024 Dec 12;11:1505655. doi: 10.3389/fnut.2024.1505655 (PMC11670750; doi:10.3389/fnut.2024.1505655)
Supplement: Supplementary file 7 [file Image_1.pdf]

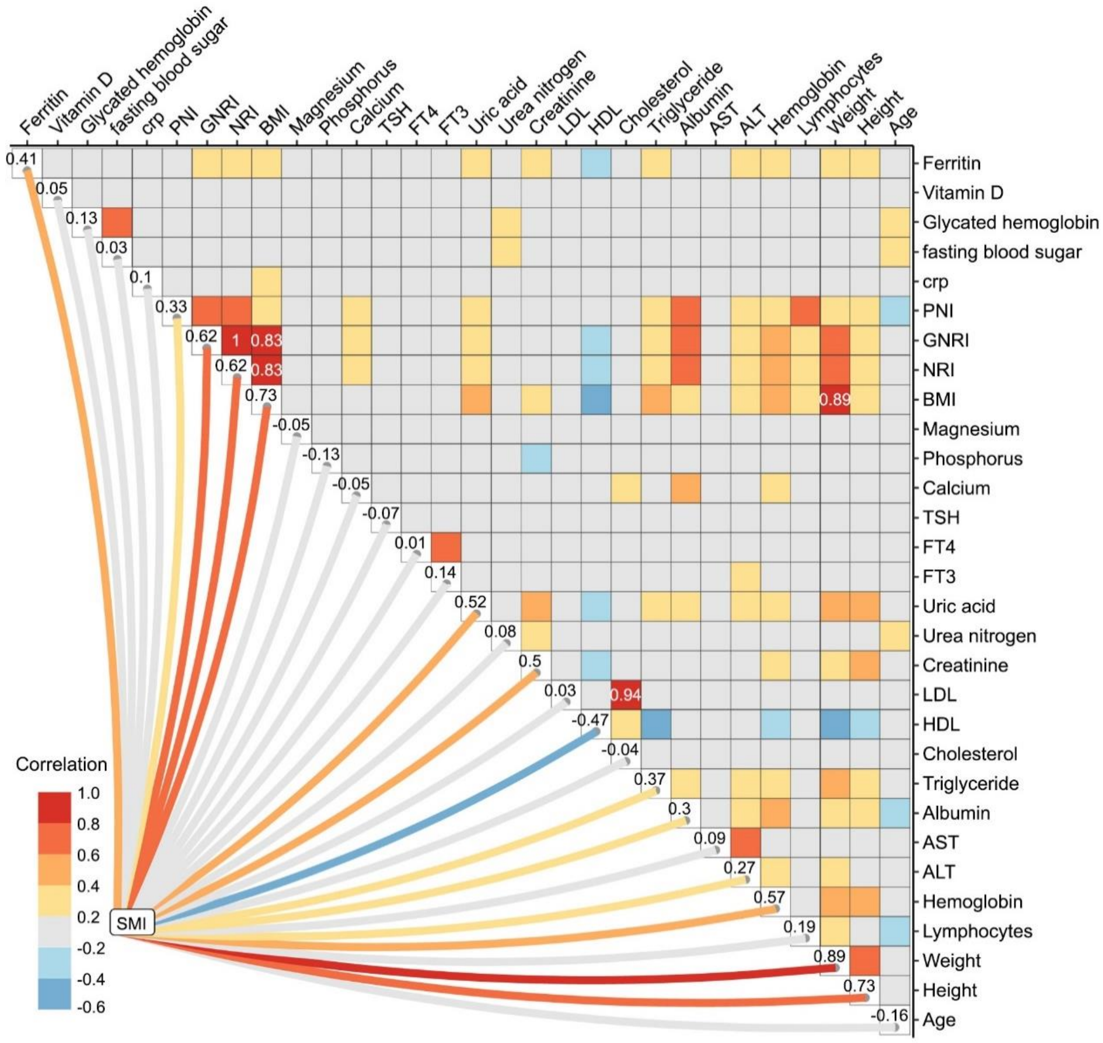

Supplementary Figure 1 Correlation analysis between Skeletal Muscle Index (SMI) and clinical indicators

The left-side lines represent the correlation coefficients between SMI and each clinical indicator, while the right-side shows the autocorrelation of each indicator.
