# Supplementary material for: Development and validation of a predicative model for identifying sarcopenia in Chinese adults using nutrition indicators (AHLC)
Source: Front Nutr. 2024 Dec 12;11:1505655. doi: 10.3389/fnut.2024.1505655 (PMC11670750; doi:10.3389/fnut.2024.1505655)
Supplement: Supplementary file 8 [file Image_2.pdf]

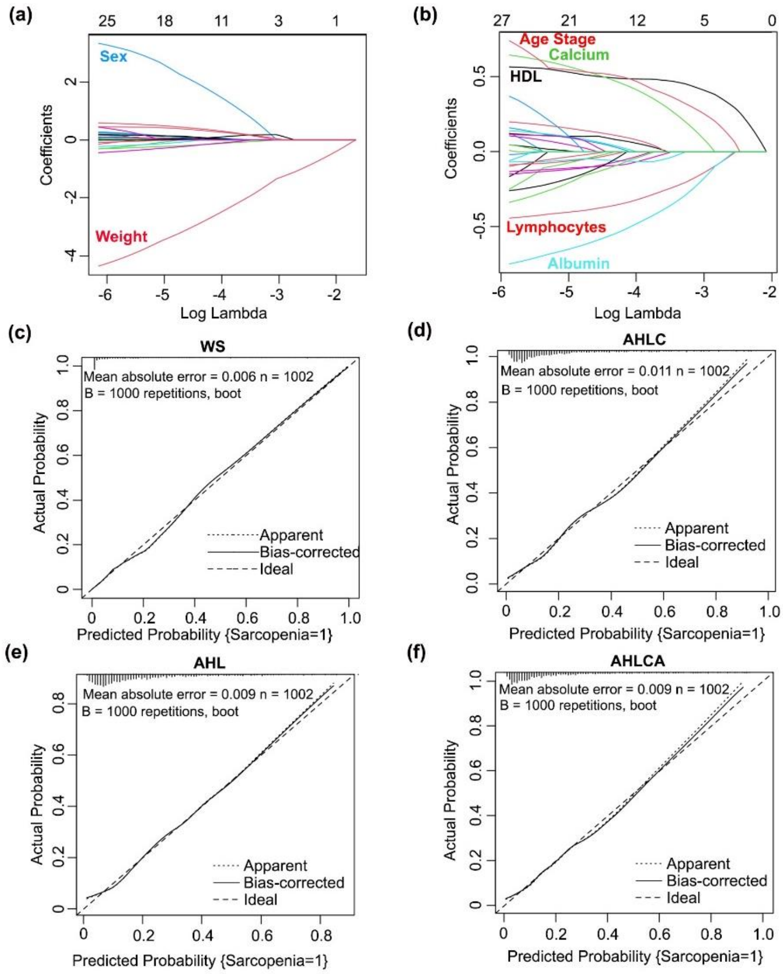

Supplementary Figure 2 Variable Screening and Model Calibration Curve Analysis

(A and B) The importance of variables was assessed using lasso regression analysis with all variables, including weight-related indicators (A) as well as after removing weight-related indicators. (C-F) The calibration curves of the constructed models are shown for WS (C), AHLC (D). AHL (E) and AHLCA (F). WS (Weight + Sex), AHL (Albumin + HDL + Lymphocytes), AHLC (Albumin + HDL + Lymphocytes + Calcium), AHLCA (Albumin + HDL + Lymphocytes + Calcium + Age Stage).
